# The French Experience of Pharmacists and CAR T‐Cells: A Study of the French Society of Oncology Pharmacy (SFPO)

**DOI:** 10.1002/hon.70144

**Published:** 2025-10-22

**Authors:** Vérane Schwiertz, Romain de Jorna, Adeline Quintard, Marie‐Antoinette Lester, Marine Pinturaud, Nicolas Cormier, Elise D’Huart, Emmanuelle Fougereau, Muriel Carvalho, Benjamin Sourisseau, Pauline Gueneau, Mathieu Wasiak, Alexia Jouvance, Muriel Paul, Régine Chevrier, Bertrand Pourroy, Jean‐Louis Cazin, Florence Ranchon, Isabelle Madelaine‐Chambrin, Catherine Rioufol

**Affiliations:** ^1^ SFPO Société Française de Pharmacie Oncologique Paris France; ^2^ Pharmacie Groupement Hospitalier Sud Hospices Civils de Lyon Lyon France; ^3^ Pharmacie Hôpital Saint‐Louis Assistance Publique des Hôpitaux de Paris Paris France; ^4^ Pharmacie CHU de Montpellier Montpellier France; ^5^ Pharmacie CHU de Rennes Rennes France; ^6^ Pharmacie CHRU de Lille Lille France; ^7^ Pharmacie CHU de Nantes Nantes France; ^8^ Pharmacie CHRU de Nancy Nancy France; ^9^ Pharmacie Institut Paoli Calmette Marseille France; ^10^ Pharmacie Hôpitaux Universitaires Henri Mondor Assistance Publique des Hôpitaux de Paris Créteil France; ^11^ Pharmacie CHU de Bordeaux Bordeaux France; ^12^ Pharmacie CHU de Dijon Dijon France; ^13^ Pharmacie CHU de Clermont‐Ferrand Clermont‐Ferrand France; ^14^ Pharmacie Centre de lutte contre le cancer Jean Perrin Clermont‐Ferrand France; ^15^ Pharmacie Assistance Publique de Marseille Marseille France; ^16^ Faculté de Pharmacie de Lille UFR 3S Université de Lille Lille France; ^17^ Université Claude Bernard Lyon I ‐ EA 3738 CICLY Lyon France

**Keywords:** CAR T‐cells, patient pathway, pharmaceutical circuit, vein‐to‐vein time

## Abstract

The aim of this study was to describe the initial 3‐year experience in vein‐to‐vein time for axi‐cel therapy and the role of pharmacists in the first recruiting French centers. Retrospective observational data were collected for vein‐to‐vein time for commercial axi‐cel after ≥ 2 lines of systemic therapy between January 2019 and December 2021 in the first 12 authorized French centers. Hospital pharmacists used a circuit database to ensure the prospective traceability at all steps. Totally 501 of the 562 intention‐to‐treat registrations on the database for cytapheresis (89,1%) led to the infusion of axi‐cel. Median vein‐to‐vein time was shortened by 4 days. This was mainly due to tightening the interval from apheresis to release. The 36‐day median vein‐to‐vein time achieved after 3 years' experience should be compared to the 29–34 days reported in Canada, the USA and Israel, where manufacturing sites are geographically closer to hospital centers than they are in France. The top 5 recruiting centers had the shortest vein‐to‐vein times. This French experience may serve as a model for other European centers, notably as regards deployment of pharmacists to improve the patient pathway with CAR T‐cells and other gene and cellular therapies.

## 1 Introduction

CAR‐T cells (Chimeric Antigen Receptor T cells) have emerged as a significant therapeutic and organizational innovation in the treatment of refractory or relapsed hematological malignancies. Nearly 10 years after the two phase 1–2 non‐comparative studies JULIET [1]and ZUMA‐1 [2], leading respectively to approval of tisagenlecleucel (tisa‐cel) and axicabtagene ciloleucel (axi‐cel), CAR‐T cells are now used in an increasing number of indications and increasingly early lines of treatment [3, 4, 5, 6]. However, their novel and complex manufacturing process, based on ex vivo genetic transformation of the patient's own T lymphocytes after apheresis, involves costs around €320,000 per patient, a time‐consuming drug circuit and a complex patient pathway from intention to treat inclusion to infusion. Vein‐to‐vein time needs to be as short as possible, as eligible patients, already in relapse or refractory to previous treatments, have potentially life‐threatening prognoses.

To address these issues, CAR‐T cell therapy deployment strategy in France has used several levers: feedback from North American centers, two French laws fixing conditions for CAR T‐cell treatment in French centers [7, 8], and the setting up of the nationwide CAR‐T cell registry DESCART by the LYSA‐LYSARC network with other associated academic groups (GRAALL, IFM, SFCE, FILO and the scientific society SFGM‐TC), centralizing data collection for both academic and health authority purposes, and enabling coverage by the French public health system [9]. In addition, the health centers with the greatest recruitment of patients with hematological malignancies have been selected for deployment of CAR‐T cell therapy, with exchange of experience organized via scientific societies (The French Society of Oncology Pharmacy SFPO, the European Hematology Association EHA, the European Society for Bone and Marrow Transplantation EBMT). The European Union and French regulations referred to CAR T‐cells as Advanced Therapy Medicinal Products (ATMPs) and categorized them into gene therapy medicines. This medicines status requires hospital pharmacists to be responsible for the pharmaceutical circuit of CAR T‐cells, including ordering and receiving, cryogenic storage, thawing, and dispensing [10, 11, 12, 13]. Feedback from the French experience must be shared to improve organization. Based on the axi‐cel circuit model, the present study aimed to describe the initial 3‐year experience in vein‐to‐vein time for patients with hematological malignancy, and the role of pharmacists in the first recruiting French centers.

## 2 Methods

The present analysis includes the first 3 years' experience of the first 12 recruiting centers in France authorized for CAR‐T cell treatment that had treated patients with axi‐cel between January 1, 2019 and December 31, 2021: university hospitals in Paris (Saint‐Louis and Mondor hospitals, Assistance Publique ‐ Hôpitaux de Paris), Lyon (Lyon Sud hospital, Hospices Civils de Lyon), Rennes, Montpellier, Nancy, Bordeaux, Lille, Nantes, Dijon, Clermont, and the cancer center of Marseille (Institut Paoli‐Calmettes). For three university hospitals (Bordeaux, Clermont and Mondor), axi‐cel treatments began in 2020.

Centers with their first patient treated with axi‐cel after December 31, 2021 did not participate in the study.

Data were prospectively registered upon intention‐to‐treat (ITT) on the website set up by the pharmaceutical manufacturer to ensure the traceability of all steps of the circuit. Depending on the local organization of the centers, cytapheresis was performed in the cytapheresis unit of the hospital or by the French blood bank, either on site or outside the hospital. The fresh collected apheresis materials were shipped to the manufacturing facility in the U.S until June 2020 and thereafter to Amsterdam. The finished batches underwent quality control, allowing delivery by the manufacturing facility and then shipment to the hospital pharmacy of each center. Each bag of axi‐cel underwent a conformity check by the pharmaceutical team, followed by cryogenic storage. In some centers, the hospital pharmacy subcontracted storage to an existing on‐site cell therapy unit.

Each hospital pharmacy secured the CAR‐T cell circuit in its own center, except the Mondor center subcontracted the pharmaceutical circuit out to the Saint‐Louis center for ordering, receiving and cryogenic storage only. The Saint‐Louis center is organized as a CAR‐T cell hub for neighboring centers, ensuring supply, cryogenic storage, and delivery on the day of administration.

On reception of the bag, the pharmaceutical team ensured the conformity check, including checking the cryo‐shipper, opening the metal cassette to fully inspect the frozen cell product and checking the CAR‐T cell label (patient identity and drug identity). The CAR T‐cells were then stored under cryogenic conditions. A first green light was given by the hematologist, and lymphodepletion chemotherapy was prepared in the pharmaceutical preparation unit and then administered to the patient. Within 5–7 days, a second medical green light was given for administration of the CAR‐T cells; the axi‐cel bag was thawed by the pharmaceutical team and dispensed to the hematology unit for infusion. Additional interventions included preparing the lymphodepletion chemotherapy, dispensing interleukin 6 receptor inhibitor tocilizumab to manage any cytokine release syndrome (CRS), informing the patient about the drug, and assisting the nurse during infusion.

Reception of any out‐of‐specification (OOS) CAR T‐cells, without clinical risk (i.e., with a low number of viable cells), was managed by manufacturer and the hospital team.

Apheresis and infusion rates were calculated by center, by year, and for the overall study period.

Apheresis rate was defined as number of apheresis out of the total number of ITT‐registrations.

Infusion rate was defined as number of axi‐cel infusions out of the total number of apheresis (“infusion rate/apheresed”) or registrations on the manufacturer's website (“infusion rate/registered”).

All the consecutive steps of the circuit were tracked on the manufacturer platform website: registration of ITT, apheresis, pharmaceutical release of the batch in the manufacturer's production site, delivery to the administration center, and infusion. The number of ITT‐registrations, apheresis and infusions and the number of delivered CAR‐T cells were entered on the website by each center, along with times for each step. Reasons for treatment cancellation were not tracked but were collected retrospectively from the quality assurance files.

Apheresis was considered as baseline (T0). Turnaround time was defined as the total time from apheresis to infusion.

A survey sent out to pharmacists collected the number infusions of all CAR‐T cells (axi‐cel or other, in standard care, early access or clinical trials) in each center, the key‐steps of the care pathway and CAR‐T cell circuit (location of apheresis and cryostorage), and pharmacy team human resources for managing CAR‐T cells.

## 3 Results

### 3.1 Center Organization Results

#### 3.1.1 Organization of the First 12 French Centers Authorized for CAR‐T Cells

Eight of the 12 centers had complete on‐site organization, with cytapheresis, cryostorage and infusion; the other 4 had partial organization, with off‐site apheresis for 3 centers, off‐site cryostorage for 1 and both apheresis and cryostorage off site for 1. Cryostorage was available in the pharmacy premises in 4 centers, or contracted out to the cell‐therapy unit in 5 or to the biology laboratory in 1. The centers with only partial on‐site organization were among those with the lowest recruitment except in 1 case, and time intervals seemed to be longer; this finding deserves further investigation in a larger sample of centers.

#### 3.1.2 Pharmaceutical Interventions in the French Experience

The survey showed that hospital pharmacists specialized in cancer care were involved at each step of the CAR‐T cell and patient pathway: participation in the multidisciplinary concertation meeting to validate the eligibility of the patient and the indication, then ordering the drug, reception and conformity check, handling and cryogenic storage, thawing, sterile preparation whenever necessary, then dispensation to the hematology unit. In addition, pharmacists prepare the bridging chemotherapy and lymphodepletion chemotherapy. The hospital pharmacist was involved at the time of infusion, to respond to any request such as for an extra device or administration advice to nurses, and then participates in follow‐up, ensuring immediate availability of symptomatic drugs in case of adverse events such as cytokine release syndrome requiring interleukin 6 receptor inhibitor antibodies. Pharmaceutical interviews with the patient and a community‐hospital link with the general practitioner and community pharmacist can be set up to secure follow‐up. During all these stages, the pharmaceutical team worked closely with the hospital hematologist and nurses, which included participation in multidisciplinary team meetings and a close relationship with the pharmaceutical manufacturer to take account of inherent production times.

In addition, the pharmacy teams were involved in managing occasional problems during the 3‐year study period: a delivery on which the identification label on the bag had become unstuck, and leakage in one bag, found on thawing; in agreement with the manufacturer, two bags of CAR T‐cells were destroyed by the pharmacist because of death of the patients after reception.

#### 3.1.3 Pharmaceutic Human Resources

The pharmacy teams began the CAR‐T cell activity with additional resources of around 1 pharmacist per center in the 3 centers with the greatest recruitment, treating 40–50 patients per year during the study period. One had 1.7 pharmacists, and the others 0.5, in relation to their activity. In 2 centers with low recruitment, CAR‐T cell activity began with no extra human resources. There were no changes in resources during the study period.

## 4 Pathway Results

From January 1, 2019 to December 31, 2021, totally 1049 CAR‐T cells were infused as approved indication of hematologic malignancies in the participating centers, including 17% in clinical trials.

626 ITT‐registrations were entered on the website for an indication for axi‐cel as approved at the time of study: relapsed or refractory diffuse large B‐cell lymphoma (DLBCL) after ≥ 2 lines of systemic therapy, DLBCL arising from follicular lymphoma, high‐grade B‐cell lymphoma, or primary mediastinal large B‐cell lymphoma. 562 apheresis (89.8% of ITT) then 501 infusions of axi‐cel were registred (89.1% of apheresis and 80.0% of all ITT‐registrations).

Rates of apheresis, infusion and treatment cancellation are shown per year and for the whole study period in Table 1.

**TABLE 1:** Rates of ITT, apheresis and infusions per year during whole study period.

	2019	2020	2021	Total (2019–2021)
Number of centers	10	12	12	12
Circuit step:				
ITT‐registrations				
Number	184	228	214	626
Mean/center ± standard deviation [range]	18 ± 13 [1–40]	19 ± 8 [8–34]	18 ± 10 [4–37]	52 ± 26 [15–95]
Median	21.5	16	18	51
Apheresis				
Number	163	213	186	562
Mean/center ± standard deviation [range]	16 ± 11 [1–30]	18 ± 8 [8–31]	16 ± 8 [3–32]	47 ± 23 [14–85]
Median	20	15	16	46
% apheresis/registration	88.59	93.42	86.92	89.78
Axi‐cel production and delivery				
Number	153	197	177	527
Mean/center ± standard deviation [range]	15 ± 10 [1–30]	16 ± 7 [8–29]	15 ± 8 [3–32]	44 ± 22 [13–78]
Median	18	14	14	42
% delivery/apheresis	93.87	92.49	95.16	93.77
Infusions				
Number	145	188	168	501
Mean/center ± standard deviation [range]	15 ± 10 [1–28]	16 ± 7 [8–28]	14 ± 8 [3–31]	42 ± 22 [11–77]
Median	16	13	12	40
% Administration/registration	78.80	82.46	78.50	80.03
% Administration/apheresis	88.96	88.26	90.32	89.15
% Administration/production	94.77	95.43	94.92	95.07
Treatment cancellation				
Number	39	40	46	125
Mean/center ± standard deviation [range]	3 ± 4 [0–12]	3 ± 2 [0–6]	4 ± 2 [0–7]	10 ± 6 [0–24]
Median	2	4	5	11
% cancellation/registration	21.20	17.54	21.49	19.97
% Cancellation/apheresis	23.9	18.8	24.73	22.24

The treatment was canceled in 125 cases (19.97%) between ITT‐registration on the website and infusion. Reasons were given by pharmacists in 87 cases and were most frequently related to the clinical condition (53.60%). Other reasons concerned pathway coordination (10.40%) or manufacturing (5.60%). Cancellations occurred mainly between registration and apheresis (26.40%), or between delivery and infusion (20.80%). Table 2 shows reasons for non‐infusion per step. Pharmacists declared delivery of 10 out‐of‐specifications (OOS) CAR‐T cell bags during the study period, 7 of which were infused despite a lower rate of viable cells.

**TABLE 2:** Reasons for non‐infusion per step (*n* = 125).

Reasons for treatment cancellation	Step			
	ITT→ apheresis	Apheresis → delivery	Delivery→ infusion	Total
Health status	26	18	23	67
Change in health status	26	18	23	67
Manufacturing	—	7	—	7
Lab refusal of apheresis quality		1		1
Production failure		3		3
OOS		3		3
Pathway coordination	7	3	3	13
Cancellation by center	2	3	3	8
Other	5			4
Total/step	33	28	26	87/125
Not documented by the center				38

The mean number of axi‐cel infusions per year and per center varied between centers, from 5 to 26. The 5 centers with the strongest recruitment per year (16–26 infusions per year) were also those with the strongest recruitment over the study period as a whole (49–77): university hospitals Lyon‐Sud, Saint‐Louis, Montpellier, Rennes and Lille. These were among the first to open in France, in 2018 or early 2019. After apheresis, infusion rates were 0.91–0.94, systematically above the overall average of 0.89.

### 4.1 Step Time Results

**FIGURE 1:**
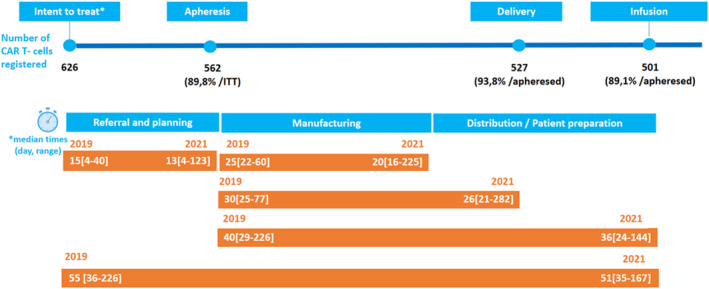
Patient journey intended for axi‐cel: Percentage of patients who underwent apheresis compared to ITT, percentage of patients for whom CAR T‐cells were delivered and then injected compared to patients who underwent apheresis ‐ Median time (median number of days, min, max) for each stage of the process. *registred into the axi‐cel website.

Figure 1 shows apheresis, delivery and infusion rates, and median times with a range [min‐max] of each step of the pathway.

The median manufacturing time has been reduced by 5 days during the s study period, which may partly explain the reduction of the same number of days in the median vein‐to‐vein time.

Of the 462 patients treated during the study period, 300 were treated by the 5 centers with the highest recruitment rates (60 ± 10.63 patients per center vs. 23.14 ± 10.63 patients in the 7 other centers). The 5 centers with the strongest recruitment showed shorter median intervals than overall, for all years and all steps, with two exceptions. In one center, the median turnaround time from apheresis to infusion was longer in 2019, due to too early of ITT‐registration on the website, with apheresis not yet in place; in another, the median time from apheresis to reception of axi‐cel was longer during the overall study period, because reception was limited to 2 days a week for reasons of human resources and organization, in agreement with the Hematology unit and therefore without clinical impact. An extremely long manufacturing time (225 days) was observed in one case. This production time includes the production of a first CAR T‐cells that was not infused due to OOS, plus the production time for the second CAR T‐cells which was finally not infused due to a change in the patient's health status.

## 5 Discussion

The present study demonstrates the rapidity and reliability of the pharmaceutical circuit and the patient's pathway experience in France. This is the first multicenter study to put together infusion rates, axi‐cel access times and organizational parameters over a period of 3 consecutive years. This was made possible by the coordination of the multidisciplinary care teams in the first 12 authorized French centers. CAR‐T cell deployment strategy in France is based on French regulations [7, 8] that determine the conditions for health center authorization for use of CAR‐T cells, and on the DESCART registry that ensures feedback to the health authorities [9]. Centers with the strongest recruitment of patients with hematological malignancies were selected to begin deployment, and their experience was shared via the relevant scientific societies. The high level of recruitment of these first centers continued over the following years, despite new centers opening, confirming the need for this treatment nationwide. The high apheresis and infusion rates, both close to 90%, and their stability over 3 consecutive launch years testify to the positive impact of learning within the centers and to effective transfer of know‐how. According to previous results [14] in Europe, infusion rates seemed not to change during the Covid‐19 pandemic, although management of cancer patients was slowed down [15]. Moreover, the mean infusion rates in the 5 centers with strongest recruitment were similar to those reported elsewhere, such as in Canadian centers, which reported infusion rates close to 92% in apheresed patients [14, 16].

*Shorter vein‐to‐vein time from cytapheresis to infusion in patients treated by CAR‐T cells for relapsed or refractory DLBCL has become a burning issue, since it was associated with favorable complete response rates and overall survival [17]. Canadian centers reported median times from apheresis to delivery of 17–21 days [2, 16]. The French centers were at a greater distance from the production site than in Canada, which may explain the longer median time of 26 days reported in the present study, which was comparable to the 25 days reported in European center [14]. Notwithstanding this distance, the 36‐day median vein‐to‐vein time achieved after 3 years' experience can be compared to the 29–34 days reported in Canada [16], the USA and Israel [14], where manufacturing sites are geographically closer to hospital centers than they are in France. Moreover, the median vein‐to‐vein time in the French centers was very similar from the European experience of 37.9 days in a comparable geographical setting [17]. In addition, median vein‐to‐vein time improved by 4 days during the first 3 years of the French experience. This was mainly due to the reduction in median manufacturing time. The change in the production site for axi‐cel from US to Amsterdam during the study inevitably reduced transport times to French centers compared to production in the US at the start of the study. This was also due to tightening the interval from each step to the next, including faster access to apheresis, greater fluidity in supply, shorter order times and improved communication with the manufacturer thanks to pharmacists using the circuit database. All these actions still need to be improved. Precise application of patient eligibility criteria and successful efforts to improve medical organization and circuits are certainly among the strengths of the French centers. Interestingly, the top 5 recruiting centers had the shortest vein‐to‐vein times, underlining the importance of training and sharing experience. In addition, well‐designed center organization, notably regarding the pharmaceutic circuit, help shorten vein‐to‐vein time. Most French centers set up a complete on‐site circuit, with cytapheresis, cryostorage and infusion. The pharmaceutical circuit of CAR‐T cells was a lever, with dedicated approved cryostorage premises, within the pharmacy unit itself or else with an arrangement with the cell therapy unit, except in 2 low‐recruitment centers. When fully on‐site organization is not feasible, a system with a central “hub” pharmacy provides a solution.

The pharmacist's involvement at each step of the patient pathway, from supply to patient education, was also helped by allocation of resources, averaging around 1 full‐time pharmacist in the most strongly recruiting centers, which were managing 40–50 patients a year at the time of this study.

Other factors also contribute to timely initiation of CAR‐T cell treatment, such as referring appropriate patients to treatment centers, availability of manufacturing slots, and prompt commencement of lymphodepletion chemotherapy for CAR‐T infusion after product delivery. Apheresis rates and times need further improving, as patients' health status worsens, with risk of loss of chance. Some factors may continue to be optimized as French centers get more experience in treating eligible patients with CAR‐T cell therapy, in earlier treatment lines. Practices continue to be improved, and excellence is demonstrated by recent data from the French DESCART nationwide registry for 309 patients in the second‐line axi‐cel early access program between July 2022 and August 2023, with vein‐to‐vein time of 36 days and a 55‐day interval between the tumor board CAR‐T cell treatment decision and actual infusion, or “brain‐to‐vein time” [18].

## 6 Limitations

The study concerned only axi‐cel treatment in validated indications, whereas, during the study period, patients were also treated with tisa‐cel, a different approved autologous anti‐CD19 CAR‐T cell therapy, as well as other CAR‐T cells in clinical trials. Also, outlying median times can be found in particular situations, and should be studied on a case‐by‐case basis.

## 7 Conclusion

These results highlight the French experience of vein‐to‐vein time for patients treated with CAR‐T cells. This French experience may serve as a model for other European centers, notably as regards deployment of pharmacists to improve the patient pathway with CAR‐T cells and other gene and cell therapies.

## Ethics Statement

This study focused on the drug circuit and did not require the norms required for research involving human participants. Human ethics and informed consent was not applicable.

## Conflicts of Interest

The authors declare no conflicts of interest.

## Peer Review

The peer review history for this article is available at https://www.webofscience.com/api/gateway/wos/peer-review/10.1002/hon.70144.

## Data Availability

The authors have nothing to report.
